# Kinetic characterization of *trans-*proteolytic activity of Chikungunya virus capsid protease and development of a FRET-based HTS assay

**DOI:** 10.1038/srep14753

**Published:** 2015-10-06

**Authors:** Megha Aggarwal, Rajesh Sharma, Pravindra Kumar, Manmohan Parida, Shailly Tomar

**Affiliations:** 1 Department of Biotechnology, Indian Institute of Technology Roorkee, Roorkee-247667, India; 2 Division of Virology, Defence Research and Development Establishment, Gwalior 474002, India

## Abstract

Chikungunya virus (CHIKV) capsid protein (CVCP) is a serine protease that possesses *cis*-proteolytic activity essential for the structural polyprotein processing and plays a key role in the virus life cycle. CHIKV being an emerging arthropod-borne pathogenic virus, is a public health concern worldwide. No vaccines or specific antiviral treatment is currently available for chikungunya disease. Thus, it is important to develop inhibitors against CHIKV enzymes to block key steps in viral reproduction. In view of this, CVCP was produced recombinantly and purified to homogeneity. A fluorescence resonance energy transfer (FRET)-based proteolytic assay was developed for high throughput screening (HTS). A FRET peptide substrate (DABCYL-GAEEWSLAIE-EDANS) derived from the cleavage site present in the structural polyprotein of CVCP was used. The assay with a Z’ factor of 0.64 and coefficient of variation (CV) is 8.68% can be adapted to high throughput format for automated screening of chemical libraries to identify CVCP specific protease inhibitors. Kinetic parameters K_m_ and k_cat_/K_m_ estimated using FRET assay were 1.26 ± 0.34 μM and 1.11 × 10^3^ M^−1^ sec^−1^ respectively. The availability of active recombinant CVCP and cost effective fluorogenic peptide based *in vitro* FRET assay may serve as the basis for therapeutics development against CHIKV.

Chikungunya virus infection has been characterized by high fever, muscle pain, gastrointestinal troubles, headache, rashes, eye and neurological problems. The virus is transmitted through mosquitoes *Aedes aegypti* and *Aedes albopictus*. The incubation period for Chikungunya ranges from 2–12 days followed by an acute phase that lasts for days to weeks. In some cases, severe Chikungunya infection leads to chronic disease characterized by persistent arthralgia from weeks to years^1^. CHIKV was first isolated in 1953 in Tanzania^2^ and afterwards it became an epidemic in Asia and Africa from the period 1954 to 2000^3^,^4^. Subsequently, major outbreak started in 2004 with an epidemic in Kenya^5^ that lead to the spread of CHIKV in Madagascar, Comoro, Mayonette and La Réunion islands of Indian Ocean, India, West Africa and South-East Asia^6^,^7^,^8^,^9^. According of the data provided by Centers for Disease Control and Prevention (CDC), more than 60 countries and territories are listed where Chikungunya cases have been reported (http://www.cdc.gov/chikungunya/geo/index.html).

Besides Chikungunya fever, the infection also includes lymphopenia, lethal hepatitis, encephalitis and neonatal encephalopathy^10^,^11^. Due to A226V mutation in E1 glycoprotein (involved in viral fusion) of CHIKV, an additional mosquito vector *Aedes albopictus* was observed during the La Réunion epidemic^12^. Thus, high pathogenicity and increase in the disease-transmitting vector makes CHIKV crisis more intense and needs immediate attention towards its eradication or virus specific treatment. Recently, a number of attempts have been made to develop vaccines or antivirals against CHIKV infection. The virus like particles (VLPs) of Chikungunya virus has been proposed to be used as vaccine against CHIKV^13^. Also, the immunoglobulins transfer and IFN-α treatment have provided some hint for the prevention of CHIKV infection^14^,^15^. Despite of the vaccine preparation trials, a number of antivirals such as ribavirin (a broad spectrum antiviral) and chloroquine (considered as to block virus entry and maturation) were tested for antiviral activity *in vivo* with no positive results^16^,^17^,^18^,^19^. However, chloroquine was found to have beneficial effect on chronic arthralgia caused by the infection of CHIKV^20^.

Chikungunya virus belongs to the alphavirus genus of *Togaviridae* family having a positive-sense single-stranded RNA genome. The genomic RNA consists of two open reading frames (ORFs) that encode for the non-structural polyprotein and the structural polyprotein of the virus. The structural polyprotein is translated from the 26S subgenomic RNA and consists of CP-E3-E2-6K-E1^21^. The capsid protein (CP) is present at the N-terminus and consists of two domains: the amino terminal and the carboxyl terminal domains. The amino-terminal domain is involved in capsid-capsid interaction to form the nucleocapsid core assembly^22^,^23^. Also, this domain is involved in capsid-RNA interaction to encapsidate the genome^24^,^25^,^26^. Thus, the amino-terminal domain of CP plays important role in the virus life cycle. However, this N-terminal domain is highly disordered and does not form structural architecture as described through X-ray crystallography and biochemical studies^27^,^28^,^29^. The carboxyl terminal domain of CP acts as a serine protease, which undergoes autoproteolysis to separate CP from the rest of the structural polyprotein^28^,^30^,^31^,^32^. Additionally, the carboxyl terminal protease domain contains a hydrophobic pocket through which it interacts with the viral glycoproteins during virus budding^33^,^34^,^35^. This hydrophobic pocket has also been proposed to bind the N-terminal arm of the neighboring capsid molecule during nucleocapsid core assembly^29^.

The C-terminal protease domain of alphavirus CP has a chymotrypsin-like serine protease scaffold that contains the catalytic triad residues Ser, His and Asp similar to other serine proteases^36^. The GDSG sequence motif, which contains the active site residue Ser, is conserved in all the serine proteases. All alphavirus capsid proteases cleave the scissile bond between the conserved Trp-Ser residues corresponding to the *cis* auto-cleavage site present at the C-terminus of CP (CVCP residues: W261-S262) (Fig. 1). CP detaches itself from the structural polyprotein after auto-proteolytic cleavage and the conserved P_1_ Trp residue at the carboxyl-terminus of CP remains bound to the S_1_ substrate specificity pocket near the active site^28^,^29^,^35^,^37^. Tryptophan bound in the specificity pocket makes the active site inaccessible to the substrate for *trans*-proteolytic cleavage and inhibits the *trans*-protease activity of the CP. Consequently, the alphavirus capsid protease is active only for one proteolytic reaction in the life cycle of virus. Further studies conducted on Aura virus capsid protease (AVCP) and Semliki Forest virus capsid protease (SFCP) demonstrate that the deletion of conserved C-terminal tryptophan leads to the restoration of the proteolytic activity of alphavirus CP^38^,^39^. The activity of SFCP was determined using nitrophenyl esters of tryptophan and tyrosine as the substrates. However, recently, the *trans*-proteolytic activity of the C-terminal truncated AVCP and the crystal structure of the active form of AVCP have been determined^39^.

The *cis*-autoproteolytic cleavage of CP in alphaviruses including Chikungunya virus is the first step in structural polyprotein processing. Therefore, the capsid protease activity in alphaviruses is considered as a potential antiviral drug target. Hence, it is necessary to characterize the proteolytic activity of CVCP and develop an *in vitro* protease assay for screening potential inhibitors. The FRET based assay has been proven to be an important tool for the analysis of enzymatic activities of proteases from different pathogens and for the development of high throughput assay for inhibitor screening^40^,^41^,^42^. The protease inhibitors against various viral proteases including HCV (hepatitis C virus) NS3/4A protease and 3C proteases from severe acute respiratory syndrome (SARS) coronavirus; and bacterial SUMO proteases have been identified through FRET based HTS assay^42^,^43^,^44^.

In this report, we have successfully cloned the gene encoding CVCP through molecular cloning of cDNA in expression vector, expressed the protein in soluble form using *E. coli* and purified to homogeneity. The *trans*-protease activity of CVCP has been determined using highly sensitive fluorogenic peptide substrate derived from the natural cleavage site sequence. Additionally, the kinetic parameters for CVCP *trans*-protease activity were calculated using the FRET based assay. Some known protease inhibitors and other compounds were screened for the antiviral activity. The influence of pH, NaCl and glycerol concentrations were observed on the CVCP activity. The results provide the standard conditions for the *trans*-proteolytic activity assay for CVCP, which could be adopted for the HTS of CVCP inhibitors.

## Results

### Purification of active and inactive CVCP

After autoproteolysis and the release of CP from structural polyprotein, Trp261 remains bound near the active site and makes the protein further inactive for either *cis* or *trans* proteolytic action. However, previous studies on alphavirus CP indicate that the activity of CP is restored on removal of the conserved C-terminal tryptophan residue^38^,^39^. So, in order to evaluate the enzymatic activity of CVCP, both inactive and active proteins were expressed and purified. Firstly, the protease domain of CVCP was cloned into pET28c vector to form the expression plasmid pET28c-CVCP. The inactive protein contains the full protease domain (106-261 residues). Truncation of the last two residues including C-terminal Trp261 from the inactive construct results in generation of the active form of CVCP (106-259 residues). The optimization of expression conditions was done for both the proteins to get good amount of soluble protein. After optimization of induction temperature, induction time and IPTG concentration, both the proteins having the 6X-His tag at the N-terminus were expressed in the soluble form.

The soluble proteins were purified by IMAC (immobilized metal assisted chromatography) and size exclusion chromatography. In Ni-NTA column chromatography, the protein was eluted by increasing concentration of imidazole. A single band was observed on SDS-PAGE for both the purified proteins confirming the protein homogeneity (Fig. 2). The protein band was visible at ~17 kDa on SDS-PAGE which was also confirmed by gel filtration chromatography by comparing and calculating the molecular weight using standard molecular weight markers. Thus, it is confirmed that both active and inactive CVCP exist as monomer in solution.

### *In vitro trans*-protease assay of CVCP

A highly sensitive FRET based assay was used for determining the *trans*-proteolytic activity of CVCP. The *in vitro trans*-protease activity of CVCP was monitored using the peptide substrate containing 4-(4-dimethylaminophenyl-azo)benzoic acid (DABCYL) and 5-[(2-aminoethyl)amino]naphthalene-1-sulfonic acid (EDANS) at the N- and C-terminus of the peptide respectively. This pair demonstrates FRET in which EDANS acts as fluorophore and DABCYL as quencher that quenches the fluorescence produced by EDANS. In the presence of protease, the peptide gets cleaved, which separates the two fragments and reduces the FRET signal. The active CVCP exhibits the efficient cleavage of the substrate peptide, which separates EDANS (fluorophore) and DABCYL (quencher) resulting in the increased fluorescence signal. The fluorescence was quenched before the proteolytic cleavage due to the presence of DABCYL within a particular distance range from the fluorophore EDANS (Fig. 3A). The proteolytic reaction was carried out in 20 mM HEPES buffer (pH 7.0) in cuvette (1 ml reaction volume) as well as in 96-well plate (100 μl reaction volume). The substrate was added to start the reaction and the fluorescence enhancement was recorded with time (Fig. 3B). The relative fluorescence units were calculated for all the readings. Figure 3B shows the typical fluorescence profile for the hydrolysis of the substrate. A control reaction was also performed using the same reaction without enzyme, which did not show change in the fluorescence intensity (data not shown).

### Determination of kinetic parameters

For the calculation of kinetic parameters, Vi of the enzyme at different substrate concentrations was determined (Fig. 3C). All the values were normalized by subtracting the readings obtained with the same reaction having no enzyme. The fluorescence extinction coefficient (FEC) was calculated to be 35,215 RFU/μM for the FRET substrate. This FEC value was used to determine the amount of the product formation in each reaction. The kinetic parameters K_m_ and k_cat_/K_m_ were calculated as 1.26 ± 0.34 μM and 1.11 × 10^3^ M^−1^ sec^−1^ respectively (Fig. 3D). The inactive CVCP was used as a negative control in proteolytic activity assay and as expected, it showed insignificant increase in the fluorescence signal (data not shown).

### Validation of FRET assay for HTS (Z’ factor analysis)

The assay was validated statistically by determining the Z’ factor and the coefficient of variation (CV). The Z’ factor is used for the assessment of the efficiency of HTS assay. It can be defined as the degree of separation between the mean values for the positive control and the mean value of the background signal. The Z’ factor with value greater than 0.5 has been considered as an indicator of an authentic and veritable screening assay. The CV of less than 10% represents the less variation within the groups of positive and negative controls. The Z’ factor for the CVCP was calculated as 0.64 using the FRET peptide substrate, which confirms the high sensitivity and efficiency of the assay. The scatter plot distribution of the group reads for both the positive and negative controls is shown in Fig. 4. The value of Z’ factor suggests high quality of the assay with high signal to noise ratio. The degree of variability is 8.68% in the positive control group. The Z’ factor and the CV reveal the reliability of the present FRET based assay in HTS of inhibitors against CVCP.

### Inhibitor screening assay

The C-terminus Trp261, which remains bound to the specificity pocket of the inactive protein after *cis* autoproteolysis, makes CVCP inactive for further proteolytic action. Considering this, we screened few compounds and peptides (tryptophan, tryptamine, EW and EWS), and some serine protease inhibitors [(phenylmethanesulfonyl fluoride (PMSF), benzamidine, N-p-Tosyl-L-phenylalanine chloromethyl ketone (TPCK) and 3-acetylindole] for the inhibition of CVCP activity. After the calculation of the cleaved product formed, the comparison was made between the reactions with and without the compound. The CVCP was found resistant to the tryptophan-based compounds, peptides and serine protease inhibitors. It is completely resistant to tryptophan, tryptamine, EWS peptide and PMSF even at their high concentrations (100 μM). However, peptide EW could show 30% reduction in the activity of CVCP at the concentration of 50 μM. Other protease inhibitors benzamidine, TPCK and 3-acetylindole also showed less than 40% decrease in enzyme activity at 80 μM concentrations. Thus, the compounds or peptides tested did not show significant inhibitory activity against CVCP. The proteolytic inhibitory assay was tested using TPCK and chymotrypsin. The positive control with TPCK showed expected inhibitory activity against chymotrypsin with the inhibition constant, Ki = 16.5 μM.

### Effect of pH and NaCl on the enzymatic activity of CVCP

To further characterize the CVCP activity, we examined the pH profile of the enzyme activity. The effect of different buffers with different pH values has been observed on the CVCP activity. The Vi was calculated at all different pHs and plotted against the buffer pH. The graph shows a regular pattern of enzymatic reaction, which is optimum at pH 7.0 (Fig. 5A). Thus, the optimal pH for *trans*-proteolytic activity of CVCP is near the physiological pH 7.0 that mimics the intracellular environment inside the mammalian cell where it undergoes *cis*-proteolytic cleavage. Furthermore, the titration experiment for NaCl demonstrates the effect of increasing salt concentration on CVCP activity. The influence of NaCl concentration on the activity was studied using different NaCl concentrations (25, 50, 100, 200, 300 and 500 mM) in the assay buffer. The increase in the enzymatic activity was observed from 0 to 0.1 M NaCl concentration while afterwards from 0.1 to 0.5 M a gradual decrease in the activity was observed. The enzyme shows the maximum activity at 100 mM NaCl concentration (Fig. 5B). The results indicate the ionic strength dependent variations in CVCP activity.

### Influence of glycerol on enzyme activity

The effect of anti-chaotropic agent glycerol on the enzyme activity is also observed and the CVCP is found to be susceptible for the inhibition with increasing glycerol concentration. Different concentrations of the glycerol (0 to 50%) (v/v) were used and the reaction velocity was measured. At 10% glycerol, negligible increase in the activity is found, however slight increase was noticed at 20% concentration of glycerol. However, further increase in the glycerol concentrations resulted in the decrease of enzymatic activity and at 50% glycerol concentration, almost no activity is detected (Fig. 6A). The results indicate that up to 20% glycerol can be used in the reaction buffer; afterwards it inhibits the catalytic activity of the enzyme.

## Discussion

Chikungunya cases have increased drastically in last few years, which results in ravaging outbreaks. A very less number of studies have been performed till date that aimed for antivirals development against CHIKV. The non-infectious CHIKV replicon having reporter gene and the cell based phenotypic assay for nsP2 inhibitors of CHIKV have also opened up the possibilities for antiviral screening^45^,^46^. However, the follow up studies are missing. Also, the lack of an *in vitro* assay for antiviral screening studies promotes us towards more detailed understanding and development of newer methods for anti-CHIKV compounds screening. A few antiviral drugs have been developed in the last decade^47^, however, viruses are evolving rapidly and more are emerging in different areas. There is a lack of effective therapies against very important viruses. The re-emergence of CHIKV as an epidemic in a number of different regions^4^,^5^,^6^, the addition in the mosquito vector due to the evolution of virus (mutation in E1 glycoprotein)^12^ and the pathogenicity of virus including persistent arthralgia^1^ makes it a significant pathogen which needs proper attention. Hence, there is the necessity of anti-CHIKV drug development and efficient therapeutic protocol manifestation. Though the mortality rate for Chikungunya infection is not so high, the high evolution rate in the genome of CHIKV encourages us for the development of new promising antivirals and therapeutics against CHIKV.

Our experiments show the presence of CVCP as a monomer for both active and inactive CVCP in solution as also described for the AVCP^27^. It confirms that alphavirus CP shows the *trans* proteolytic activity in the monomeric form itself. Capsid protease represents very significant target for anti-CHIKV drug development as involved in the very first step of structural polyprotein processing. Fluorogenic substrates are used for the high throughput compound screening of a number of viral proteases such as SARS coronavirus 3CLpro protease, West Nile virus Serine Protease, HIV (Human immunodeficiency virus) protease, Dengue NS2B-NS3 protease, HCV (hepatitis C virus) NS3/4A protease^48^,^49^,^50^,^51^,^52^. This technique is very simple and highly sensitive; hence provides a tool for HTS of antivirals. Moreover, the *in vitro* assay is very convenient and does not need BSL2 or BSL3 containment.

In the current study, we focused on the *trans*-proteolytic activity assay of CVCP using FRET based fluorogenic peptide substrate. In the continuous fluorescence assay, there is a continuous increase in the relative fluorescence units over time. The time dependent fluorescence enhancement reveals that the truncated purified CVCP is *trans* catalytically active. For the kinetic studies, the assay was performed with the increasing concentration of substrate. The increase in the velocity occurs with increase in the substrate concentration until it gets saturated. The kinetic parameters for the active CVCP were found as K_m_ = 1.26 ± 0.34 μM and k_cat_/K_m_ = 1.11 × 10^3^ M^−1^ sec^−1^.

The FRET based proteolytic assay has also been checked if it can be used in the high throughput format and for the screening of the compounds against CVCP. For the general assessment of the assay quality and reliability, the Z’ factor and the coefficient of variation were determined in a 96 well plate and found to be 0.64 and 8.68% respectively. As demonstrated by the values of Z’ factor and CV in this study, the FRET based *in vitro* proteolytic assay of CVCP is highly convenient and suitable for the HTS of the compounds. The assay might provide a perfect approach to explore first generation CVCP inhibitors. Using a fluorescence plate reader, this assay can be easily performed in a 96- and 384- well plate format for high throughput inhibitor screening against the CVCP. This high throughput protease assay is simple, convenient and cost effective. Few compounds were screened for the inhibition of the CVCP activity. The tryptophan, tryptamine compounds and peptides EW, EWS could not inhibit CVCP activity. Additionally, different serine protease inhibitors PMSF, benzamidine, TPCK and 3-acetylindole also did not seem to act as potent inhibitors of CVCP. This is consistent with the previous reports for the *cis*-autoproteolytic activity of CVCP and the *trans*-esterase activity of SFCP where complete resistance to the standard serine protease inhibitors was observed. The *cis*-autoproteolytic activity of the CVCP was not inhibited by serine protease inhibitor AEBSF (4-(2-Aminoethyl) benzene sulfonyl fluoride hydrochloride)^53^. Additionally, the *trans* esterase activity of the SFCP did not show inhibition in the presence of the protease inhibitor PMSF, chloromethylketones (N-benzyloxycarbonyl-leucine-tyrosine-chloromethylketon and N-benzyloxycarbonylphenylalanine-chloromethylketone) and N-acetyl-glutamate-glutamate-tryptophan p-nitro-anilide^38^. Hence, it is possible that substrate specificity pockets of CVCP have a distinct conformation as seen in the crystal structure of active AVCP (PDB ID: 4UON) in which Pro215 obtrude to block Trp267 position^39^.

The influence of various conditions on the proteolytic activity of the active CVCP has also been determined. In this respect, pH range, different NaCl and glycerol concentrations were used for the measurement of the activity and the relative activity was calculated. The optimum enzyme activity was found at pH 7.0 and 100 mM NaCl concentration. The optimum pH is neutral pH which corresponds to the pH of the mosquito saliva and the human cells. Thus, the optimal activity occurs at the physiological pH 7.0 to match with the host cell pH. The effect of pH on the enzyme activity might be due to the conformational variations in the enzyme structure at different pHs. Additionally, the response of the enzymatic activity with increasing ionic strength indicates the dependence of the protease structure on the salt concentration. The significant increase in enzymatic activity up to 100 mM NaCl concentration might be due to the stabilization of the enzyme with increasing ionic strength, however, the decrease in activity above 100 mM NaCl might be the result of alteration in molecular interaction at the active site or the specificity pocket at high NaCl concentration. The increase in ionic strength might disrupt ionic and polar interactions in the structure of CVCP leading to conformational changes in the active site.

The anti-chaotropic agent glycerol also affects enzyme function. Glycerol can be added up to 20% to the reaction buffer in the CVCP protease assay reaction. In general, glycerol acts as the stabilizing agent for the protein and is not expected to reduce the enzymatic activity. Interestingly, at 50% concentration of the glycerol in the reaction buffer, there is complete loss of the CVCP enzymatic activity. It might be due to the binding of glycerol molecule in the S_1_ specificity pocket of CVCP that could block entry of the substrate inside the pocket and thus, inhibit catalytic reaction. To explore this possibility, the three dimensional structure of active CVCP was predicted by homology modeling based on the crystal structure of active AVCP (PDB ID: 4UON). CVCP molecular model consists of a chymotrypsin-like serine protease fold with two subdomains and the active site is located at the interface of the subdomains (Fig. 6B). In CVCP, the active site is contributed by His139, Asp161 and Ser213 residues. The S_1_ specificity pocket is present near the active site to which the conserved P_1_ substrate residue Trp261 is expected to bind. Furthermore, the superimposition of the homology model of active CVCP in complex with glycerol onto the native AVCP (inactive AVCP, PDB ID: 4AGK) (RMSD = 0.127 Å) shows interaction of glycerol molecule exactly at the same position where conserved P_1_ tryptophan residue binds in the S_1_ specificity pocket (Fig. 6C). The S_1_ specificity pocket of CVCP includes residues Trp189, Ile203 to Ser213, Ile227 to Asn233, and Thr238 to Leu240. These residues create a hydrophobic environment for the binding of substrate. The glycerol molecule fits perfectly inside this hydrophobic pocket in active CVCP structure. The hydroxyl groups of glycerol molecule show interactions with the backbone of Lys209 and also with the active site residue Ser213. In the crystal structure of active AVCP (PDB ID: 4UON), glycerol was used as a cryoprotectant during data collection and was assumed to stabilize the protein by binding to the S_1_ pocket^39^. Hence, it can be concluded from the activity assay results coupled with the structural comparison and analysis that at high concentrations glycerol might be competing with the substrate peptide for binding to the S_1_ specificity pocket of CVCP.

It can be inferred that the monomeric form of truncated CVCP is *trans*-proteolytically active and the described FRET based assay can be used in HTS of compounds against CVCP. High throughput screening of small molecule compound libraries against CVCP will be the next essential step for identification of potential CVCP inhibitors that are likely to inhibit the first step in structural polyprotein processing. Thus, the compounds interfering with the proteolytic activity of CVCP might be the potential candidate drugs against CHIKV infection. Hence, the study makes the *trans*-protease activity assay for CVCP available and provides the channel for HTS of compound libraries against alphavirus capsid protease that will be beneficial in combating the emerging pathogenic Chikungunya virus.

## Methods

### Preparation of CHIKV stocks

An Indian isolate of Chikungunya virus, DRDE-06 (Genbank accession no: EF-210157.2) belonging to East Central and South African (ECSA) genotype maintained at Virology Division, DRDE, Gwalior was used in the present study. The C6/36 cells (*Aedes albopictus* larval cell line) was obtained from National Center for Cell Science (NCCS), Pune. The cells were cultured in Eagle’s minimum essential medium (EMEM) supplemented with 10% tryptose phosphate broth (TPB), 10% fetal bovine serum (FBS), 80 U gentamicin, 2 mM L-glutamine and 1.1 g/L sodium bicarbonate (NaHCO_3_). Briefly, monolayer of cells were grown at 90% confluency and washed with plain medium prior to infection. The virus was allowed to adsorb to the cells for 1 h at 37 °C in serum-free medium. Following adsorption, the inoculum was removed by washing once with Dulbecco’s phosphate-buffered saline (D-PBS) and replenished with maintenance medium (EMEM-TPB with 2% FBS). The culture medium was harvested on appearance of cytopathic effects after 48 h post infection. Aliquots were stored in a −80 °C freezer.

### RNA extraction

Viral RNA was isolated from 140 μl of the clarified infected culture supernatant using QIAamp viral RNA mini kit (Qiagen, Germany) according to manufacturer’s protocol. The viral RNA was quantified, aliquoted and stored at −80 °C up to use.

### Molecular cloning and construction of expression plasmid

The obtained RNA was denatured at 70 °C for 5 min and chilled on ice. The first strand cDNA was synthesized using oligo(dT)_30_ primer with M-MulV reverse transcriptase at 37 °C for 1 h. The primers for recombinant DNA cloning were designed according to the sequence provided in NCBI (National Center for Biotechnology Information) (Table 1).

CHIKV DNA fragment (~3.7 kbp) encoding the structural polyprotein was amplified using the synthesized cDNA as a template. The oligonucleotides F1 and R1 were used for the amplification of structural polyprotein. The amplified product was again subjected to polymerase chain reaction (PCR) with the primers F2 and R2 having *NdeI* and *BamHI* restriction enzymes sites. The PCR product was purified with the use of PCR purification kit from Qiagen and restriction digestion of the purified PCR product was done. The pET28c vector was digested using the same *NdeI* and *BamHI* restriction enzymes. Both the digested products were subjected to agarose gel electrophoresis and the digested bands were excised. The DNA was eluted from these excised bands of agarose gel using DNA gel extraction kit (Qiagen, USA). The two DNA fragments were ligated overnight at 15 °C with T4 DNA ligase. The ligated mixture was transformed into *E. coli* cells *DH5α* (DE3) and the culture was spread on Luria Bertani (LB) agar plate having 50 μg/ml kanamycin. The plate was incubated overnight at 37 °C. The colonies were picked and screened for the presence of structural polyprotein encoding gene insert.

The confirmed constructs containing the gene encoding structural polyprotein of CHIKV was used as a template for the amplification of both the inactive CVCP (residues 106-261) and the active C-terminal truncated CVCP (residues 106-259). The amplification of CVCP was carried out using oligonucleotides: F3 and R3 for inactive CVCP, and F3 and R4 for active CVCP having *NdeI* and *XhoI* sites. Using the same protocol described above, the PCR amplified products were subcloned into pET28c vector containing Tobacco Etch Virus (TEV) protease cleavage site instead of thrombin. Positive clones containing the insert were identified by PCR and restriction enzyme digestion of isolated plasmids. Further, DNA sequencing of the isolated plasmids was done to confirm the presence of gene fragments encoding inactive and active CVCP. The N-terminal Tobacco Etch Virus (TEV) protease cleavable His_6_-tag was incorporated in both the constructs to facilitate protein purification using Ni^+^^2^ affinity chromatography.

### Expression of both inactive and active CVCP

The optimization of expression conditions for both CVCPs was carried out to produce the proteins in soluble form. Both the plasmid constructs were transformed into the *E. coli* strain *Rossetta* (DE3) and plated on LB agar plates containing kanamycin (50 μg/ml) and chloramphenicol (35 μg/ml). The plates were incubated overnight at 37 °C and a single colony was picked and grown overnight in LB broth supplemented with appropriate antibiotics for both the constructs. Using the inoculums from overnight primary cultures, 1L secondary cultures were grown in 2 L conical flasks at 37 °C. For inactive CVCP, secondary culture was grown till the OD at 600 nm (OD_600_) reaches to ~0.8 and afterwards the protein expression was induced using 0.4 mM isopropyl *β*-D-1-thiogalactopyranoside (IPTG). After induction, culture was grown at 37 °C for 4 h. For the expression of active CVCP, the secondary culture was grown till the OD_600_ reaches ~0.5 and then the culture was transferred to 18 °C. The expression of active CVCP was induced using 0.4 mM IPTG at an OD_600_ of ~0.7. After induction, the culture was further grown for ~12 h at 18 °C. The cells were harvested by centrifugation and analyzed on 15% SDS-PAGE (Sodium dodecyl sulfate-polyacrylamide gel electrophoresis) to confirm the protein expression and solubility.

### Purification of active and inactive CVCP

Purification of both the proteins was done using cell pellet from 1L culture. The pellets were re-suspended in 25 ml of binding buffer (50 mM Tris-HCl pH 7.6, 15 mM imidazole and 100 mM NaCl) on ice. The re-suspended cells were disrupted using the cell disruptor (Constant Systems Ltd, Daventry, England) and centrifuged at 4 °C at 16,000 × g to separate the supernatant and pellet. The Ni-NTA (Nitrilotriacetic acid) agarose beads (Qiagen, USA) that were pre-equilibrated with the binding buffer were loaded with the clarified supernatant and incubation was done for half an hour at 4 °C. The N-terminal His-tagged proteins were eluted using 250 mM Imidazole. Pure protein fractions eluted from the affinity chromatography column were pooled and His-tag was cleaved by overnight incubation of purified proteins with TEV protease during dialysis against the dialysis buffer (50 mM Tris-HCl pH 7.6, 20 mM NaCl) at 4 °C. After cleavage of His-tag, protein samples were reloaded onto Ni-NTA column to remove His-tagged TEV protease and uncleaved His-tagged protein. The flow-through containing purified proteins without His-tag was concentrated to ~5 mg/ml using a 3 kDa cutoff Amicon Ultra-15 concentrator (Millipore, Bedford, Massachusetts, USA). Concentrated proteins were loaded onto HiLoad 16/60 Superdex 75 pg size-exclusion chromatography column (GE Healthcare) using 50 mM Tris-HCl pH 7.6, 20 mM NaCl as buffer (filtered and degassed). ÄKTA purifier (GE Healthcare) purification system kept at 4 °C operated at a flow rate of 0.5 ml/min was used to conduct the gel-filtration chromatography step. Fractions of the major peaks were collected and analyzed using 15% SDS-PAGE. Gel-filtration fractions containing protein of interest were pooled and again concentrated to ~5 mg/ml. The molecular weight marker proteins bovine serum albumin (66 kDa), ovalbumin (45 kDa), trypsin (23 kDa) and lysozyme (14 kDa) were loaded and run on the size-exclusion HiLoad 16/60 Superdex 75 column to determine the void volume of the column and to construct the standard curve. Further, the standard curve was used for estimating the molecular weight of the purified proteins. The UV absorbance spectroscopy at a wavelength of 280 nm using an extinction coefficient of 23,950 M^−1^ cm^−1^ for inactive CVCP and 18,450 M^−1^ cm^−1^ for active CVCP was used for the estimation of the concentration of both the proteins.

### Fluorogenic peptide substrate

**Figure 1:**
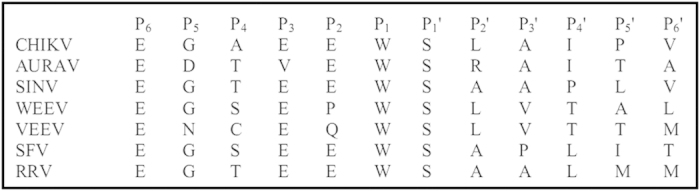
The peptide sequence of the CP from different alphaviruses showing the residues P_6_-P_6’_ surrounding the scissile bond residues Trp and Ser.

**Figure 2:**
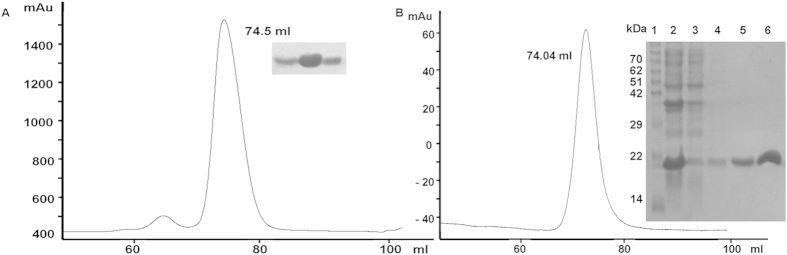
SDS-PAGE and gel filtration profile of CVCP. Both (**A**) inactive and (**B**) active CVCP shows the protein purified to homogeneity. The size-exclusion chromatography results suggest the monomeric nature of both the proteins. Lane 1, molecular-weight markers (kDa); lane 2, pellet containing insoluble protein fraction; lane 3, supernatant containing soluble protein fraction; lane 4–6, purified CVCP.

**Figure 3:**
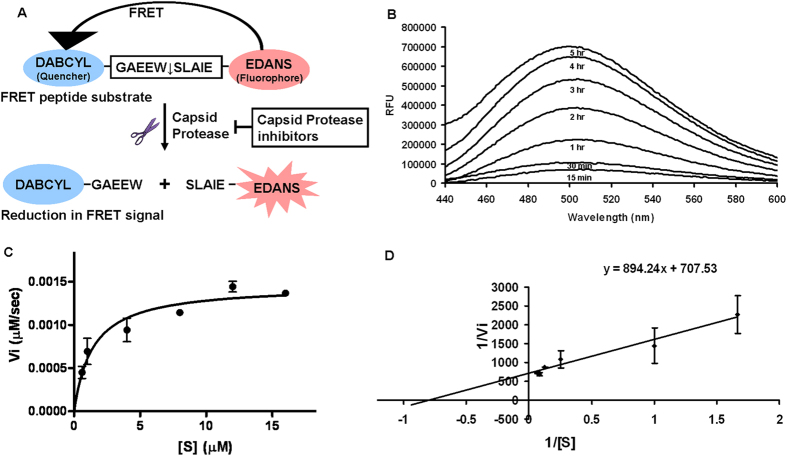
*In vitro* FRET based assay. (**A**) A FRET based protease assay has been developed for the determination of CVCP activity. The DABCYL (Quencher) and EDANS (fluorophore) are attached at the N and C terminus of the peptide respectively. The donor EDANS produces the signal upon excitation that gets quenched by DABCYL and thus shows FRET. In the presence of protease, the cleavage takes place and the fluorophore and quencher gets separated from each other, which results in the reduction of FRET signal. In the presence of protease inhibitors, the FRET should remain unaltered. (**B**) *In vitro trans* proteolytic assay was performed in 20 mM HEPES pH 7.0 buffer and the fluorescence was measured at different time points. The fluorescence was measured till 5 hrs and the increase in fluorescence was observed. All the values are the average of triplicate data. The values were normalized using the reaction performed in the similar conditions with no enzyme. (**C**) To perform the kinetic studies of CVCP, different concentrations of the substrate ranging from 0.6 μM to 16 μM were used and initial velocities were calculated for all substrate concentrations. The kinetic data were fitted into the Michealis-Menten equation. (**D**) The lineweaver-burk plot was formed and the intercept and slope were calculated according to the equation y = mx + c. From these, the values of V_max_ and K_m_ were determined and the k_cat_ was also calculated by dividing V_max_ with the enzyme concentration.

**Figure 4:**
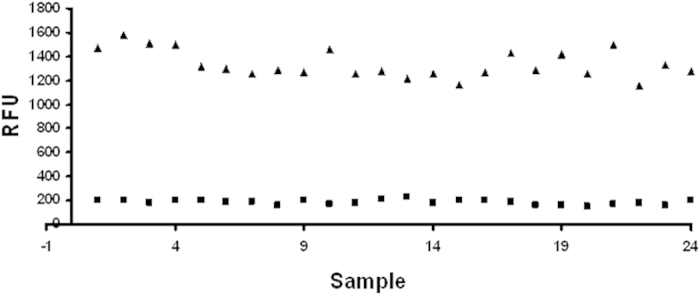
Z’ factor analysis. The scatter plot showing the positive control (triangle shaped) and negative control (square shaped) data for the Z’ factor calculation. 24 samples were used for computing the means and standard deviations. The experiment was performed in triplicate.

**Figure 5:**
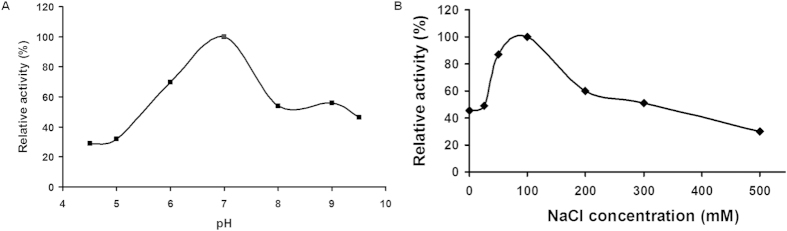
The effect of pH and NaCl concentration on the enzymatic activity was observed. (**A**) Using the buffers of pH ranging from 4.5 to 9.5, the pH optimum was calculated for the proteolytic activity. The relative activity was calculated at different pHs by taking the activity at pH 7.0 as 100%. (**B**) The activity at NaCl concentration of 100 mM was taken as 100% and the relative activity for other NaCl concentrations was calculated. All the readings are the average of the triplicate data and the values are normalized by using readings obtained from the reaction containing no enzyme.

**Figure 6:**
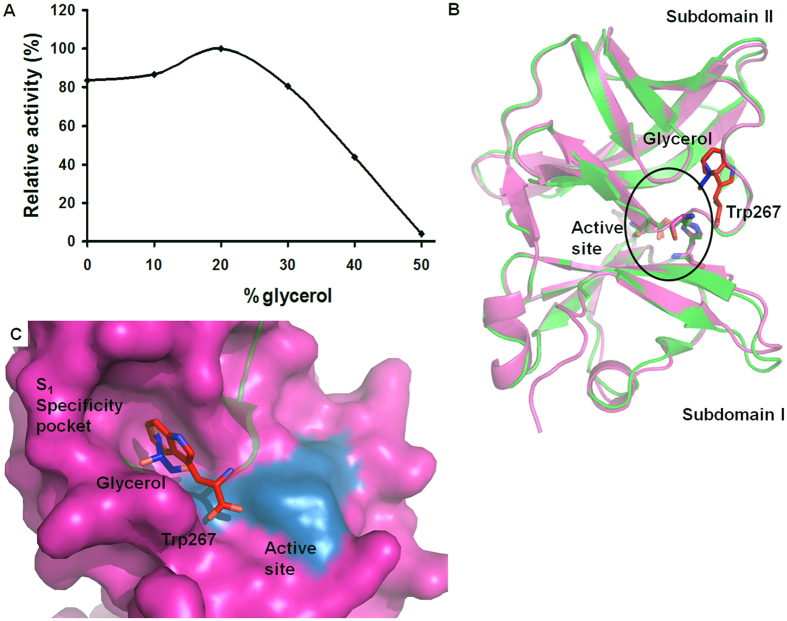
Influence of the glycerol on CVCP activity. (**A**) The effect of increase in glycerol concentration (0 to 50%) in the reaction buffer on the enzymatic activity of CVCP has been observed and the relative activity was measured by taking the activity at 20% glycerol as 100%. The graph represents the average data obtained from three experiments. (B) CVCP 3D homology model (magenta) with glycerol in the S_1_ specificity pocket was generated and superimposed on the crystal structure of AVCP with bound P_1_ residue (green) (PDB ID: 4AGK). The overall structure shows the presence of two subdomains; the active site is present at the interface of these two subdomains (active site residues are shown in sticks). The S_1_ specificity pocket is present in the vicinity of the active site (shown in circle) to which the P_1_ residue Trp267 of native AVCP binds. (**C**) The zoom-in surface view of the S_1_ pocket shows that glycerol (blue) in the CVCP structure binds incisively at the position where conserved P_1_ residue Trp (red) binds in AVCP. The active site is shown in blue color.

A FRET based peptide substrate was designed for *in vitro trans*-proteolytic activity of the CVCP. The custom synthesized peptide DABCYL-GAEEWSLAIE-EDANS was used as a substrate (Biolinkk, New Delhi, India). This substrate peptide consists of nine amino acids derived from the P_5_ to P_4_′ positions of the cleavage site of CVCP. The numbering was assigned to the substrate residues in which the P_1_ and P_1_′ positions include the residues at scissile bond. Other residues are enumerated towards the amino and carboxyl terminus respectively (Fig. 1).

### *In vitro trans*-proteolytic activity assay

The *trans*-protease activity of CVCP was measured by a FRET assay using the custom synthesized fluorogenic peptide substrate: DABCYL-GAEEWSLAIE-EDANS. The rate of CVCP proteolytic activity was determined by measuring the increase in fluorescence intensity of enzymatic reactions conducted in a 1 ml fluorescence cuvette or a 96-well black microplate (Corning Inc., USA). The reactions were performed in 20 mM HEPES, pH 7.0 with varied concentration of substrate for the kinetic measurements. Reactions were initiated by the addition of 1 μM enzyme from a concentrated stock, and were monitored continuously over a time period of approximately 15 to 300 min. All reactions were performed at 25 °C in temperature-controlled conditions. The fluorescence was monitored at 490 nm with the excitation wavelength of 340 nm using Fluorolog-3 Spectrofluorimeter (Horiba Jobin Yvon) or Cytation 3, the fluorescence microplate reader (BioTek Instruments, Inc.). The enhancement in the fluorescence readings due to the cleavage of peptide substrate leading to dissociation of FRET pair and decrease in energy transfer was observed with time. Cuvette based assays were conducted using quartz cuvettes with a pathlength of 1.00 cm in a reaction volume of 1 ml. The emission spectra were recorded and scanned from 440 to 600 nm with 1 nm bandwidth. The inner filter effect was negligible as the fluorescence measurements were carried out using low concentrations of fluorogenic peptide substrates, where the fluorescence intensity was linearly proportional to the concentration of the fluorescent peptide substrate. A reaction volume of 100 μL was used for conducting 96-well microplate assays. For 96-well plate assay, the excitation and emission wavelengths of the fluorogenic peptide substrate and the associated bandwidths for the filters were Dabcyl 340 (16) nm and Edans 535 (16) nm. Chymotrypsin was used as a positive control in the FRET assay for CVCP.

### Determination of fluorescence extinction coefficients

The FEC is used for the determination of the amount of product formation over time. To determine the FEC under certain instrument settings and assay conditions, the reaction was carried out in the assay buffer (20 mM HEPES, pH 7.0) using varied concentration of substrate (0.6–16 μM) with the use of excess CVCP. The use of excess enzyme ensures the rapid and complete cleavage of the substrate. The curve between the relative fluorescence units (RFU) (fluorescence of the reaction minus the fluorescence obtained with the reaction in the absence of enzyme) and the varying substrate concentration was plotted. The slope of the curve was calculated which is assigned as the FEC for the substrate peptide.

### Determination of reaction rates and turn over number

The initial reaction rate was calculated by performing the above-described reaction in triplicate. The rate was determined by monitoring the change in the RFU over time. The RFU was converted to the concentration of the product formed using FEC. The reaction was also carried out using the same protocol but no enzyme to determine the baseline proteolysis rate. The initial velocity at different time points was calculated and plotted against varied substrate concentrations for the determination of the kinetic parameters including V_max_, K_m_ and k_cat_/K_m_ values. The turn over number was calculated using the concentration of the capsid protease enzyme used.

### Calculation of Z’ factor and Coefficient of variation (CV)

The Z’ factor is used to evaluate the quality of the HTS assays. The Z’ factor was calculated according to the method described by Zhang *et al.*, 1999^54^. It was determined with the use of FRET substrate described above. The substrate was added to the assay buffer containing capsid protease enzyme or the assay buffer without enzyme. The fluorescence readings were measured for both positive and negative controls to calculate the standard deviations and means. The coefficient (Z’ factor) was obtained with these values and can be defined as:


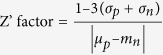


where σ_p_, σ_n_, μ_p_, and μ_n_ are the standard deviations (σ) and averages (μ) of the positive (p) and negative (n) controls respectively. The CV was calculated as the ratio of the standard deviation and average of the sample readings. This value is expressed in percentage after multiplying the ratio with 100.

### CVCP inhibition assay

Inhibitory activity of various commercially available serine protease inhibitors (PMSF, benzamidine, TPCK and 3-acetylindole), tryptophan, tryptamine (a derivative of tryptophan), and substrate-derived peptides (EW and EWS) were tested against CVCP protease. These compounds were purchased from Sigma-Aldrich (St Louis, MO) and the custom synthesized peptides were procured from Biolinkk Pvt. Ltd, Delhi. The stock solutions of 10 mM were made in 100% DMSO, and the required final concentrations of inhibitors were made by dilution in assay buffer. The concentrations of DMSO in the final reactions did not exceed 1.0% (v/v). The substrate was used at a final concentration of 1 μM to minimize the inner filter effect and to produce strong signal. The active CVCP was pre-incubated with the compounds for 30 min followed by addition of substrate peptide. The concentration of compounds used was 50–100 μM in the final assay volume of 100 μl. Enzyme reactions were performed in 96-well half area black flat bottom plates (Corning Life Sciences) at 25 °C and the fluorescence readings were taken at different time points (from 15 to 120 min). The amount of the cleaved product was calculated as described above and compared with the control reaction well without any inhibitor. The formation of the cleaved product should get reduced in the presence of potential inhibitor of the CVCP. TPCK, the known chymotrypsin inhibitor and chymotrypsin were used as positive control for enzymatic inhibition assay.

### Influence of pH, NaCl and glycerol

The effect of pH on enzymatic activity was observed using different buffers with pH ranging from 4.5 to 9.5. The buffers used for optimizing the pH range were 20 mM sodium acetate (pH 4.5, 5.0, 5.5), MES (pH 6.0, 6.5), HEPES (pH 7.0, 7.5), Tris-HCl (pH 8.0, 8.5), Bicine (pH 9.0) and Glycine-NaOH (pH 9.5). The influence of varying concentration of NaCl and glycerol on the proteolytic activity of CVCP was analyzed by performing the reaction in varying concentrations of NaCl (0 to 0.5 M) and glycerol [10 to 50% (v/v)]. The initial velocity for the enzyme was calculated in all different conditions and the relative activity was measured. All the experiments to determine the optimal assay conditions for CVCP activity were performed in triplicate.

### 3D model generation

Homology modeling was performed for active CVCP in the following steps: (a) template selection from protein data bank (PDB), (b) sequence template alignment, (c) model building, (d) refinement and validation. NCBI BLAST search tool was used to perform the template search. The crystal structure of AVCP (PDB ID: 4UON) was selected as a template with 71% sequence identity to CVCP. Clustal Omega program was used for multiple sequence alignment of the query sequence with the template sequence (http://www.ebi.ac.uk/Tools/msa/clustalo/). Further, the alignment was used to build the PIR format, alignment file as an input for MODELLER (Modeller 9.11). The ligand Glycerol (GOL) was incorporated in PIR format while generating homology model. Models were built which gave C alpha root-mean-square deviations (RMSDs) of <1 Å, from the observed structures. Several models were generated and the model with the lowest DOPE score was further evaluated using PROCHECK and ERRAT (http://nih server.mbi.ucla.edu/SAVES/). The model with the least number of residues in the disallowed region was further evaluated for energy minimization using Swiss-Pdb Viewer 4.01(http://spdbv.vital-it.ch/).

## Additional Information

**How to cite this article**: Aggarwal, M. *et al.* Kinetic characterization of *trans*-proteolytic activity of Chikungunya virus capsid protease and development of a FRET-based HTS assay. *Sci. Rep.* **5**, 14753; doi: 10.1038/srep14753 (2015).

## Figures and Tables

**Table 1:** List of the oligonucleotides used.

Name	Sequence
F1	5′- ATGGAGTTCATCCCAACCCAAACTTTTTACAATAGG AGG -3′
R1	5′- TTAGTGCCTGCTGAACGACACGCATAGCAC -3′
F2	5′- ACGAACATATGATGGAGTTCATCCCAACCCAAAC -3′
R2	5′- AAGCAGGATCCTTAGTGCCTGCTGAACGACAC -3′
F3	5′- CTGGAATTCATATGTGCATGAAAATCGAAAATGATTGTATTTTCG -3′
R3	5′- CTAGAATCTCGAGCTACCACTCTTCGGCCCCCTC -3′
R4	5′- CTAGAATCTCGAGCTATTCGGCCCCCTCGGG -3′
